# Insights into Patient Experiences with Facilitated Subcutaneous Immunoglobulin Therapy in Primary Immune Deficiency: A Prospective Observational Cohort

**DOI:** 10.1007/s10875-024-01771-0

**Published:** 2024-08-05

**Authors:** Ezgi Yalcin Gungoren, Melek Yorgun Altunbas, Ummugulsum Dikici, Zeynep Meric, Isil Eser Simsek, Ayca Kiykim, Salim Can, Esra Karabiber, Nalan Yakici, Fazil Orhan, Haluk Cokugras, Metin Aydogan, Oner Ozdemir, Sevgi Bilgic Eltan, Safa Baris, Ahmet Ozen, Elif Karakoc-Aydiner

**Affiliations:** 1 Depatment of Pediatrics, Division of Allergy and Immunology, Faculty of Medicine, Marmara University, Istanbul, Turkey; 2 Istanbul Jeffrey Modell Diagnostic and Research Center for Primary Immunodeficiencies, Istanbul, Turkey; 3 The Isil Berat Barlan Center for Translational Medicine, Division of Pediatric Allergy and Immunology, Marmara University, Istanbul, Turkey; 4 Department of Pediatrics, Division of Allergy and Immunology, Sakarya University, Training and Research Hospital, Sakarya, Turkey; 5 Depatment of Pediatrics, Division of Allergy and Immunology, Istanbul University- Cerrahpasa, Istanbul, Turkey; 6 Department of Pediatrics, Division of Allergy and Immunology, Faculty of Medicine, Kocaeli University, Kocaeli, Turkey; 7 Department of Chest Diseases, Division of Allergy and Immunology, Faculty of Medicine, Marmara University, Istanbul, Turkey; 8 Depatment of Pediatrics, Division of Allergy and Immunology, Faculty of Medicine, Karadeniz Technical University, Trabzon, Turkey

**Keywords:** fSCIG, IgRT, IVIG, Quality of Life, SCIG, Treatment Satisfaction

## Abstract

**Background:**

Immunoglobulin G replacement therapy (IgRT), intravenous (IV) and subcutaneous (SC) routes, is pivotal in treatment of primary immunodeficiencies (PID). In recent years, facilitated subcutaneous immunoglobulin (fSCIG), a combination of rHuPH20 and 10% IgG has emerged as a delivery method to combine advantages of both IV and SC.

**Method:**

In an observational prospective cohort, we investigated patient experience with fSCIG in PID patients from 5 PID centers for up to 12 months. We assessed the efficacy and safety of this treatment with patient/caregiver- and physician-reported indicators. Additionally, we analyzed patient treatment satisfaction (TSQM-9) and quality of life (QoL).

**Results:**

We enrolled 29 patients (22 pediatric and 7 adults; 14 females and 15 males; (median: 15, min–max: 2–40.9 years) who initiated fSCIG as IgRT-naive (n = 1), switched from conventional rapid-push 10% SCIG (n = 6) or IVIG (n = 22). Among the participants, 19 (65%) exhibited antibody deficiencies, 8 (27%) combined immunodeficiencies, and 2 (7%) immune dysregulations. Remarkably, targeted trough immunoglobulin G levels were achieved under all previous IgRTs as well as fSCIG. No severe systemic adverse drug reactions were documented, despite prevalent local (%86.45) and mild systemic (%26.45) adverse reactions were noted with fSCIG. Due to mild systemic symptoms, 2 patients switched from fSCIG to 10% SCIG. The patient satisfaction survey revealed a notable increase at 2-4th (p = 0.102); 5-8th (p = 0.006) and 9-12th (p < 0.001) months compared to the baseline. No significant trends were observed in QoL surveys.

**Conclusion:**

fSCIG demonstrates admissable tolerability and efficacy in managing PIDs in addition to notable increase of patients’ drug satisfaction with IgRT. The identified benefits support the continuation of this therapy despite the local reactions.

## Introduction

Immunoglobulin Replacement Therapy (IgRT) stands as the primary pharmacological intervention for a diverse spectrum of Primary Immunodeficiency (PID) disorders, boasting a history of over six decades [1]. Currently, IgRT offers two administration routes: intravenous (IV) and subcutaneous (SC) [2, 3].

Intravenous Immunoglobulin (IVIG) replacement therapy requires IV access, administration, and monitoring by healthcare professionals. It may entail systemic adverse reactions, including headaches, nausea, vomiting, rare thromboembolic events, and hypersensitivity reactions [4–6]. Conversely, Subcutaneous Immunoglobulin (SCIG) replacement therapy has gained increasing popularity, emerging as a viable alternative to IVIG. SCIG is recognized as equally effective as IVIG but triggers fewer systemic reactions, making it a crucial option for patients intolerant to IV infusion or lacking reliable venous access [4, 7]. The subcutaneous administration route allows for self-infusion at home, a practice widely acknowledged for its positive impact on health-related quality of life. However, a primary limitation of SCIG therapy lies in the inherent resistance of the extracellular matrix (ECM), constraining the volume that can be infused at a single site. Consequently, multiple infusion sites must be utilized on a weekly or biweekly basis instead of the monthly IV infusion [8–10].

There are currently various methods for Subcutaneous Immunoglobulin (SCIG) application: the conventional 10% (pump-assisted or rapid-push method), pump-assisted concentrated 20% and the facilitated SCIG infusions [1, 11, 12]. Facilitated Subcutaneous Immunoglobulin (fSCIG) is a combination of two components: recombinant human hyaluronidase (rHuPH20) and 10% human normal immunoglobulin G (IgG). rHuPH20 acts to break down hyaluronan, thereby increasing the permeability of subcutaneous tissue, which allows for the infusion of larger volumes of IgG compared to conventional subcutaneous immunoglobulin. Consequently, fSCIG can be self-administered at home every 2 to 4 weeks using a single infusion site [1, 12]. At the current time, the fSCIG option is specifically designed to combine the advantages of intravenous and subcutaneous immune globulin therapies. It allows for the administration of larger volumes of medication at a single subcutaneous site, while requiring less frequent dosing compared to other SCIG products [1, 12]. fSCIG reduces dosing frequency while maintaining the comfort of home administration and avoiding the need for intravenous infusion. The present study investigates the 12-month experience with fSCIG in a prospective observational cohort from five PID centers. The aim is to discern patients’ administration practices in real-life experience, assess the effectiveness and safety, evaluate patient satisfaction, and measure the impact on the quality of life (QoL) among individuals with PID receiving fSCIG.

## Material and Methods

In current study, PID patients from five medical centers were enrolled. The specific PID diagnosis was determined in adherence to the International Union of Immunology Societies (IUIS) classification system and The Middle East and North Africa Diagnosis and Management guidelines [13, 14]. When the patients were classified according to the IUIS 2022 classification, 19 patients had primary antibody deficiency, 4 patients had combined immunodeficiency, 4 patients had syndromic combined immunodeficiency, and 2 patients were in the immune dysregulation group. The study cohort included 1 IgRT naive PID patient treated with fSCIG due to concerns about school absenteeism, number of injection sites and infusion intervals. Twenty-eight subjects were already on IgRT and switched to fSCIG treatment from either IVIG (n = 22) or conventional rapid-push 10% SCIG (n = 6). The median duration of IgRT administration before switching to fSCIG in the other 28 patients, excluding the naive IgRT patient, was median: 2.29 (25–75%: 0.58–5.23) years.

PID patients were switched from IVIG to fSCIG due to limited venous acsess (n = 3), school/work day loss (n = 6) and recurrent hospital admissions (n = 13), whereas due to frequent injections (n = 2) and local reactions with multiple injections (n = 4) for switch from SCIG to fSCIG. Over a prospective 12-month period, we meticulously tracked the patients for treatment effectiveness, quality of life, and treatment satisfaction. Additionally, we conducted a thorough evaluation of the utilization patterns of the drug and monitored adverse reactions for each patient across a span of up to six applications.

The Marmara University Faculty of Medicine Ethics Committee approved our study protocol under a protocol number of 09.2021.1234, dated 05.11.2021. Written informed consent was obtained from all patients before initiating any study procedure. Families of each patient provided written informed consent, and all studies were conducted in accordance with the principles of the Declaration of Helsinki.

Patients were administered HyQvia® (Shire US Inc., Lexington, MA, USA), a facilitated subcutaneous immunoglobulin, with a dual vial unit containing one vial of 100 mg immunoglobulin per milliliter and one vial 160 U of recombinant human hyaluronidase (rHuPH20) per milliliter. B-Braun Perfusor® Space infusion (Braun GmbH, Frankfurt, Germany) pump was used in patients receiving fSCIG at home. The recommended dose for fSCIG is 0.4–0.8 g/kg/month, and the dosing interval to maintain stable IgG levels varies between 2 and 4 weeks as it was recommended on package insert. Previous routes of IgRT as rapid push conventional 10% SCIG applications can be administered through self-administration at home, while intravenous treatments are obligatory to be administered in a hospital setting to local medical regulations.

### Efficacy

Treatment efficacy was assessed through a comprehensive evaluation of the following parameters in annual basis before and after fSCIG teratment including the frequency of major and minor infections and infection-related hospital admissions, duration of hospital stays, and instances of school- or work-absenteeism. Moreover, quantification of IgG levels occurred at baseline (representing the trough/stable IgG values for initial IgRT) and subsequently at intervals of 2–4 months, 5–8 months, and 9–12 months. The frequency of infections and infection-related hospital admissions, duration of hospital stays, and instances of school- or work-absenteeism were noted as annual number of occasions and days.

### Subcutaneous Administration and Utilization Patterns of fSCIG

We assessed a total of 155 incidents with fSCIG applications, with specific focus on the patients’ utilization patterns. This assessment was based on data obtained from at least two consecutive infusions up to 6 occasions (dose, volume, duration, interval, administration sides, site of infusion, needle entries).

We conducted comprehensive training sessions for all patients and/or their families on the preparation and administration of fSCIG at the time of enrollment. Some individuals underwent multiple training sessions until they demonstrated competence in the application process. Once an individual was deemed proficient in preparing and administering the drug, subsequent injections were performed at home. For home-dosing, we provided educational support for injections whenever necessary. The initial administration and training sessions exhibited varying durations per patients’ needs, spanning from 30 min to 5 h. Subsequent application durations, including the preparation phase and the infusion, progressively shortened over successive applications, ranging from 30 to 90 min after the third administration.

The fSCIG dosage varied from 220 to 700 mg per kilogram per application. It's worth highlighting that we did not implement a ramp-up method in any of our patients; nearly all patients, except one, transitioned from a different mode of IgRT. The singular IgRT-naive case, diagnosed with activated PI3K delta syndrome, exhibited normal IgG values and was deemed unnecessary for a ramp-up application.

### Adverse Reactions

All adverse events occurring within the subsequent 72 h following fSCIG application were meticulously recorded, with ongoing vigilance extended up to 6 cycles of infusions. Adverse events were categorized as either local or systemic based on their proximity to the anatomical infusion site. Systemic adverse drug reactions as being hypotension, fever, urticaria, and anaphylaxis were noted in addition to local adverse reactions including skin swelling, erythema, pain, itching, and ecchymosis if happened.

### Questionnaires for Quality of Life and Treatment Satisfaction

Quality of life and treatment satisfaction questionnaires were assessed at baseline (2–4 weeks prior to first fSCIG infusion representing the initial IgRT infusions) and subsequently at intervals of 2–4 months, 5–8 months, and 9–12 months for fSCIG infusions. We employed the KINDL instrument, specifically designed for children and adolescents, as a generic tool for assessing health-related quality of life (HRQoL). Based on age brackets, two versions of KINDL were utilized: Kid-KINDL for children and Kiddo-KINDL for adolescents.

Each version comprises 24 categorical items measured on a 5-point Likert-type scale, organized into 6 dimensions. These dimensions assess physical well-being, emotional well-being, self-esteem, family, friends, and school (school or kindergarten), with each dimension consisting of 4 items. Subscale scores are independently calculated, and a total HRQoL score is derived by summing the scores of the 6 subscales. The Kid-KINDL items are rated on a 5-point Likert-type scale, ranging from 1 (never) to 5 (always). Certain items (1, 2, 3, 6, 7, 8, 15, 16, 20, and 24) with a negative orientation require reverse coding based on the statement. Subsequently, the subscale scores for each dimension are transformed to a scoring scale of 0–100 [15, 16].

The SF-36 version 1.0 is a concise questionnaire consisting of 36 items that assess eight domains related to HRQoL. These domains include physical functioning, social functioning, role limitations due to physical problems, role limitations due to emotional problems, mental health, energy and vitality, bodily pain, and general perception of health. Additionally, the SF-36 includes an item that evaluates changes in the respondent's health status over the past year. For each domain, item scores were coded, summed, and transformed into a scale ranging from 0 (worst) to 100 (best) using the standard SF-36 scoring algorithms. Furthermore, physical and mental summary component scale (PCS and MCS, respectively) scores were computed using the algorithms provided by the developers [17–19].

The Treatment Satisfaction Questionnaire for Medication 9 (TSQM-9) is a validated instrument comprising 9 items that are divided into three scales, each demonstrating robust psychometric properties in terms of reliability and validity. The first scale, consisting of questions 1–3, pertains to the efficacy of the medication. The second scale, encompassing questions 4–6, assesses the presence of convenience. The third scale, comprising questions 7–9, measures compliance with the prescribed overall satisfaction with the medication. The TSQM-9 domain scores were computed following the recommended scoring methodology as provided by the developers of the instrument, which is described in detail in previous literature. These domain scores range from 0 to 100, with higher scores indicative of greater satisfaction within the corresponding domain [20–22].

### Data Analysis

Statistical analysis was conducted for all participants including the patients switched from fSCIG to present un-biassed real-life data by using Jamovi 2.3.26 version (The Jamovi Project, Australia). When comparing two groups, the Wilcoxon test was employed when p > 0.05, whereas the Student's t-test was used when p < 0.05, and the data followed a normal distribution. In cases of multiple comparisons where the data did not follow a normal distribution, the Friedman test was employed, followed by post-hoc analyses using the Durbin-Conover method. When there are three or more independent groups, and the group distributions are not normal, the Kruskal–Wallis test is employed. The Bonferroni correction was applied when multiple comparisons were made. The figure was created by inputting data into Graphad Prism 8XML version (GraphPad Software Inc., San Diego, California) and Adobe Illustrator 25.2.1 (Adobe Inc., San Jose, California.).

## Results

The study consisted of the patients in which, 7 (24%) were adults, and 22 (76%) were children. Of these, 22 patients were undergoing Intravenous Immunoglobulin (IVIG) treatment at the time of enrollment, six were receiving conventional Subcutaneous Immunoglobulin (SCIG), and one case was IgRT-naive. Fourteen (48%) of patients were female, 15 (52%) were male, and the median age was 15 (min–max: 2–40.9) for all participants and for 22 pediatric patients as 12.6 (min–max: 2–16.4) years. The median age of onset of symptoms was 2 (min–max: 0.25–11) years. The mean age of the patients' diagnosis was 8.03 (± 4.77) years. The median diagnostic delay was 3 years (min–max: 0–13.5).

Concerning the PID category, the distribution of diagnosis was as follows: predominantly antibody deficiency accounted for 65% (19 patients), combined immunodeficiency with associated or syndromic features constituted 21% [6 patients], combined immunodeficiency alone represented 7% (2 patients), and diseases of immune dysregulation comprised the remaining 7% (2 patients). Patients' baseline IgRT dose varied between 0.45 and 0.86 g/kg/4 weeks, with infusion ranging from 5–30 days (min–max SCIG: 5–7, IVIG: 15–30). Upon initiating fSCIG, the dose of IgRT fell in the range of 0.44 to 0.70 g/kg/4 weeks, with infusion ranging from 15 to 30 days. The distribution of application intervals was as follows: 5 (3%) applications every 15 days, 27 (17%) applications every 21 days, 63 (41%) applications every 28 days, and 60 (39%) applications every 30 days. Patients receiving fSCIG administered all 155 applications of rHuPH20 and immunoglobulin with an infusion pump assistance.

### Switch from other IgRT Modes to fSCIG and Causality

Patients were switched from IVIG to fSCIG due to venous access problems in 14% (3 patients), elimination of absenteeism from school or work in 27% (6 patients), and frequent visits due to IVIG prescription and infusions at hospital in 59% (13 patients). Among the 6 patients who switched from SCIG to fSCIG, 67% (4 patients) did so due to recurrent local skin problems (pain, itching, redness) of multiple injection, while the remaining 33% (2 patients) recognized the need for less frequent dosing associated with fSCIG. The sole IgRT-naive patient preferred fSCIG to avoid school absenteeism with IV dosing.

### Patient Experience with fSCIG

We surveyed the patient's fSCIG application was obtained from at least 2 infusions from each 4 consecutive month period. In the analysis of 155 fSCIG applications among switch patients, we found that the administered dose of immunoglobulin per body weight per infusion was higher with fSCIG than with previous IgRT treatments (fSCIG vs. IVIG p = 0.029, fSCIG vs. SCIG: p < 0.001). However, there was no significant difference in total IgRT doses per month (4 weeks) (fSCIG vs. IVIG p = 0.874, fSCIG vs. SCIG: p = 1). Additionally, the volume of the injected drug in fSCIG (median: 250, min–max: 50–450 ml) was higher compared to other SCIG forms (median: 100, min–max: 50–200 ml) (p = 0.003) but not IVIG (median: 250, min–max: 50–600 ml) (p = 1) (Table 1). The volume per kg given was not correlating with age (p = 0.84).

**Table 1:** Immunoglobulin replacement therapy administration practices of patients with primary immunodeficiency before and after facilitated subcutaneous immunoglobulin

	Baseline				
	IVIG (n = 22) median min–max	SCIG (n = 6) median min–max	fSCIG (n = 155) median min–max	p-value fSCIG vs IVIG	p-value fSCIG vs SCIG
mg/kg/infusion	500 230–700	120 110–180	500 220–700	**0.029**	** < 0.001**
g/kg/4 weeks	0.50 0.45–0.86	0.51 0.47–0.77	0.53 0.38–0.80	0.874	1
g/infusion	20 5–40	10 5–20	25 5–45	0.065	**0.008**
mL/infusion	250 50–600	100 50–200	250 50–450	1	**0.003**
mL/site	250 50–600	75 45–200	250 50–450	0.969	**0.002**
number of sites	1 1–1	1 1–2	1 1–1		
infusion site per patient (antecubital/abdominal/tigh)	22/0/0	0/6/0	0/28/1		
infusion interval days	30 15–30	7 5–7	28 15–30	0.981	** < 0.001**
duration of infusion (hour)	4 1.5–6	1 1–1.5	2.5 0.5–5	** < 0.001**	** < 0.001**
needle diameter (G)	24 22–25	24 23–24	24 24–25	** < 0.001**	**0.039**
needle length (mm)	25 9–25	15.5 12–20	12 9–15	** < 0.001**	**0.004**

IVIG, intravenous immunoglobulin; SCIG, subcutaneous immunoglobulin; fSCIG, facilitated subcutaneous immunoglobulin

Data pooled from analyses of 29 patients, encompassing a total of 155 administrations, were juxtaposed with individual historical periods, denoting each patient's prior experience with immunoglobulin treatment (SCIG or IVIG), as indicated in respective columns

The interval of drug injections remained unchanged for patients switching from IVIG to fSCIG (p = 0.981). However, fewer injections were administered after switching to fSCIG (median: 28, min–max: 15–30 days) from other SCIG treatments (median: 7, min–max: 5–7 days) (p < 0.001) (Table 1).

Among the studied formulations, SCIG had the shortest duration of Ig infusion. When comparing IVIG with fSCIG, the latter showed a shorter application time (Table 1). Notably, smaller needles (both in length and gauge) were used after switching from IVIG or SCIG to fSCIG (Table 1). When examining the anatomical sites for drug application, IVIG drugs were consistently infused through the antecubital vein, while SCIG administrations were targeted at the abdominal region. Among the 155 fSCIG applications, a minor proportion (3.9%, n = 6) occurred in the thigh, with the majority (96%, n = 149) administered in the abdomen, apply 2 fingers above the imaginary transverse umbilical line. Notably, both IVIG and fSCIG drugs were administered through a single site, in contrast to previous SCIG treatments that involved two separate sites (Table 1).

### Efficacy

The baseline IgG median for the 29 patients was 988 mg/dl (25–75%: 807–1118). At 2–4 months (n = 22), the IgG median was 1060 mg/dl (25–75%: 955–1305), at 5–8 months (n = 11) the median was 1118 mg/dl (25–75%: 948–1321), and at 9–12 months (n = 8) the median was 1085 mg/dl (25–75%: 882–1204). Notably, all treatment methods, including SCIG, IVIG, and fSCIG, achieved the target trough or stable IgG levels. Statistically, no significant differences were detected among the various treatment methods (p = 0.415).

There were no observed changes in the frequency of major infectious diseases, including sepsis, meningitis, acute bacterial pneumonia/pneumonia, otitis, sinusitis, and gastroenteritis, after the initiation of fSCIG compared to the pre-treatment period. However, the frequency of minor infections, such as acute upper respiratory tract infections, demonstrated a significant decrease following fSCIG administration. Before fSCIG, the median frequency was 1 (25–75%: 0–2) ranging from 0 to 4 while after fSCIG, it reduced to 0 (25–75%: 0–0) (p = 0.035) with a range of 0–3 per year.

Moreover, the number of missed school/work days per year exhibited a noteworthy reduction following fSCIG. The median decreased from 11 days (25–75%: 5–15) before fSCIG to 0 day (25–75%: 0–2.75 days/year) after fSCIG (p = 0.036).

### Safety

**Fig. 1:**
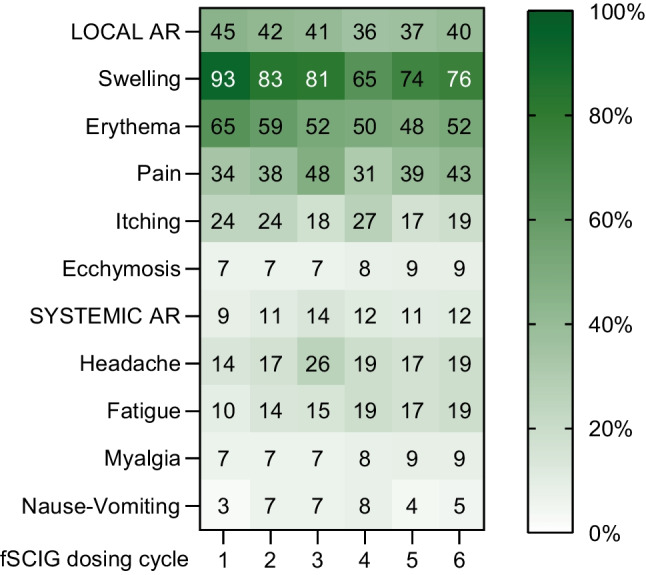
Heatmap data of for ARs related to fSCIG administration during first 6 cycles of infusions. Percentages are shown on the relevant box for each. AR, adverse reaction; fSCIG, facilitated subcutaneous immunoglobulin

We conducted a comprehensive survey to assess the occurrence of adverse events following fSCIG treatment, specifically focusing on hypotension, fever, urticaria, and anaphylaxis. Notably, none of these adverse events were observed (refer to Table 2 for details). However, local adverse reactions were documented, revealing the following distribution: swelling was observed in 79% (n = 23) of the applications, erythema in 59% (n = 85), pain during application in 42% (n = 60), itching in 24% (n = 34), and ecchymosis in 8% (n = 12). Local adverse reactions, with all instances, resolved spontaneously within 48–72 h. The mild systemic adverse reactions encompassed headache (25%), fatigue (15%), myalgia (8%), and nausea or vomiting (6%). Interestingly, in patients experiencing both local and systemic effects, there was a mild but not discernible tendency for a decrease in adverse reactions as the number of applications increased during the observation period (Fig. 1). Statistical analysis involved the use of the Friedman test, followed by post-hoc analyses using the Durbin-Conover method and significant differences were not detected in subsequent dosing cycles among local and systemic adverse reactions related to fSCIG. When evaluating local and systemic effects by age groups, no significant difference was found in the adverse reactions exhibited by the different age groups (0–6, > 6 and ≤ 12, > 12 and < 18 years, and adults).

**Table 2:** Local and systemic adverse reactions during treatment with Facilitated Subcutaneous Immunoglobulin Therapy

Dosing Cycle	#1 n = 29	#2 n = 29	#3 n = 27	#4 n = 26	#5 n = 23	#6 n = 21	TOTAL n = 155
LOCAL							
Swelling	27	24	22	17	17	16	**123**
Erythema	19	17	14	13	11	11	**85**
Pain	10	11	13	8	9	9	**60**
Itching	7	7	5	7	4	4	**34**
Ecchymosis	2	2	2	2	2	2	**12**
SYSTEMIC							
Headache	4	5	7	5	4	4	**29**
Fatigue	3	4	4	4	3	3	**21**
Myalgia	2	2	2	2	2	2	**12**
Nause-Vomiting	1	2	2	2	1	1	**9**

^#^: number of dosing cycle, n: number of application per patient

Statistical analysis involved the use of the Friedman test, followed by post-hoc analyses using the Durbin-Conover method. Significant differences were not detected in dosing cycle

Among the patients, four had a history of headaches following IVIG administration. Upon transitioning to fSCIG, two individuals persisted in experiencing headaches, managed effectively with acetaminophen premedication before infusion. In contrast, the remaining two subjects no longer reported headaches with fSCIG. Furthermore, one patient previously described vomiting and fever following IVIG infusion. Upon switching to fSCIG, these complaints subside, eliminating the necessity for premedication.

### Switch from fSCIG and Causality

During the follow-up, four patients underwent a transition from fSCIG to alternative IgRTs. Two individuals shifted to conventional 10% SCIG due to ongoing mild systemic symptoms despite premedication during fSCIG treatment. Specifically, one patient made the switch after the fourth dose of fSCIG due to ongoing nausea and headaches, while another patient transitioned after the eighth dose of fSCIG due to persistent fatigue. Additionally, two subjects opted for a switch to 20% SCIG following the sixth application of fSCIG, citing inconvenience during the pump infusion procedure of rHuPH20 and/or IgG. It's noteworthy that none of the patients discontinued fSCIG or changed to other drugs due to local adverse reactions.

### Quality of Life and Treatment Satisfaction Surveys

The KINDL scale for children and parents and the SF-36 questionnaires showed no statistically significant difference between various time points (Table 3).

**Table 3:** Assessment of Health Related Quality of Life and Treatment Satisfaction Surveys during treatment with Facilitated Subcutaneous Immunoglobulin Therapy

n median (25–75%)	Baseline	2-4th months	5-8th months	9-12th months
KINDL-Children	11 53.9 (48.1–59.1)	9 60.1 (53.6–63.7)	5 60.1 (58.3–75.3)	6 69.3 (62.9–73.8)
KINDL-Parents	12 47.5 (44.7–52.4)	10 49.3 (42.6–59.9)	6 57.3 (52.1–65.6)	6 69.3 (62.9–73.8)
SF-36	7 55.1 (53.5–68.4)	6 72.5 (57.2–83.5)	3 78.9 (54.3–81.9)	2 81.9 (80.4–83.5)
TSQM-9	16 68 (48–88.5)	18 83 (64–90)	9 88* (72–92)	8 88** (73.5–93)

KINDL, Kinder Lebensqualitätsfragebogen: Children's Quality of Life Questionnaire; SF-36, Short Form 36; TSQM-9, Treatment Satisfaction Questionnaire for Medication-9

Statistical analysis involved the use of the Friedman test, followed by post-hoc analyses using the Durbin-Conover method. Bonferroni correction has been applied. Significant differences were not detected in KINDL-Child, KINDL-Parents and SF-36 (p = 0.363, 0.120, 0.112)

In TSQM-9 (p = 0.003), however, in pairwise comparisons, there was a significant difference between *baseline vs. 5-8th months (*p* = 0.006), ** baseline vs. 9-12th months (*p* < 0.001)

**Fig. 2:**
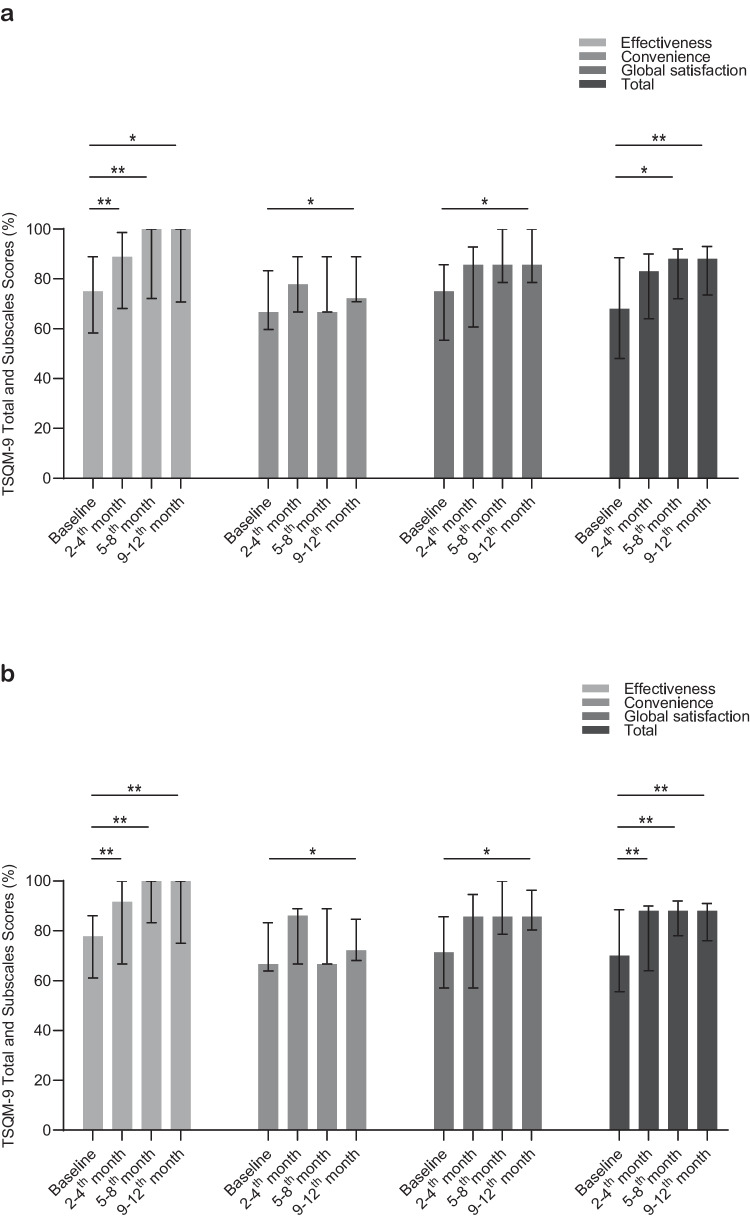
**a)** Comparison of TSQM-9 treatment satisfaction questionnaire and subdomains at baseline, 2-4th, 5-8th, and 9-12th months for IEI patients receiving fSCIG, **(b)** Comparison of TSQM-9 treatment satisfaction questionnaire and subdomains at baseline, 2-4th, 5-8th, and 9-12th months for IEI patients switched from IVIG and receiving fSCIG. The scores are presented as median (IQR 25–75%). *p < 0.05, **p < 0.001. Statistical analysis involved the use of the Friedman test, followed by post-hoc analyses using the Durbin-Conover method. Bonferroni correction has been applied. TSQM-9, Treatment Satisfaction Questionnaire for Medication-9; IEI, inborn errors of immunity; fSCIG, facilitated subcutaneous immunoglobulin; IVIG, intravenous immunoglobulin

Patients' treatment satisfaction with TSQM-9 surveys resulted in significant difference (p = 0.003) by the observation period with a increase in drug satisfaction was observed in the subsequent months; baseline (median: 68, 25–75%:48–88.5) and 2-4th months (median: 83, 25–75%: 64–90) p = 0.102; baseline vs. 5-8th months (median: 88, 25–75%: 72–92) p = 0.006, baseline vs. 9-12th months (median: 88, 25–75%: 73.5–93) p < 0.001. The effectiveness subdomain of the survey displayed significant differences, with markedly higher scores at the 2-4th months (median: 88.9; 25–75%: 68.1–98.6) (p < 0.001), 5-8th months (median: 100, 25–75%: 72.2–100) (p < 0.001), and 9-12th months (median: 100, 25–75%: 70.8–100) (p = 0.002) compared to the baseline (median: 75; 25–75%: 58.3–88.9) (Fig. 2a). According to the basal evaluation (median: 66.7; 25–75%: 59.7–83.3), the convenience subdomain had increased only in the 9-12th (median: 72.2; 25–75%: 70.8–88.9) month assessment (p = 0.021) and global satisfaction subdomain had increased only 9-12th months (median:85.7, 25–75%: 78.6–100) acording the baseline evaluation (median: 75, 25–75%: 55.4–85.7) (p = 0.048) (Table 3).

The analysis of TSQM-9 revealed a notable increase in treatment satisfaction among patients transitioning from IVIG at baseline (median: 70, 25–75%: 55.5–88.5) to fSCIG (2-4th months: median: 88, 25–75%: 64–90; 5-8th months: median: 88, 25–75%: 78–92; 9-12th months: median: 88, 25–75%: 76–91) (p = 0.001). When comparing baseline with 2-4th, 5-8th, and 9-12th months for TSQM-9, all p-values indicated significant changes (p < 0.001 for all comparisons), underscoring a consistent and meaningful improvement in treatment satisfaction over these time intervals (Fig. 2b). The effectiveness subdomain of treatment satisfaction, compared to baseline IVIG treatment, showed p < 0.001 at baseline (median:77.8, 25–75%: 61.1–86.1) vs. 2-4th (median: 91.7, 25–75%: 66.7–100), 5-8th (median: 100, 25–75%: 83.3–100), and 9-12th (median: 100, 25–75%: 75–100) months in the TSQM-9 analysis. In the effectiveness subgroup, baseline vs. 2-4th, 5-8th, and 9-12th months the respective p-values were p < 0.001. The convenience subdomain of treatment satisfaction showed statistically significant improvement (p = 0.003) at only 9-12th (median: 72.2, 25–75%: 68.1–84.7) vs baseline (median:66.7, 25–75%: 63.9–83.3). Moreover, global satisfaction subdomain of TSQM-9 survey showed similar trend from baseline (median:71.4, 25–75%: 57.1–85.7) to 9-12th months (median:85.7, 25–75%: 80.4–96.4) (p = 0.048).

## Discussion

In this multicenter, prospective, observational study, we systematically assessed the real-world experiences of patients undergoing fSCIG, placing a central focus on efficacy, safety, quality of life, and treatment satisfaction. Our analyses highlight that the subcutaneous route of administration (as opposed to IVIG) combined with less frequent dosing (compared to other SCIG therapies) positions fSCIG as a convenient method for IgRT. Beyond its convenience, our findings underscore the effectiveness and safety of fSCIG in PID patients, contributing to a positive treatment satisfaction.

The primary drawbacks of IVIG treatment, as identified by patients prompting a shift to fSCIG, included the need for hospital visits for intravenous infusions, resulting in school or work absenteeism. In line with findings from prior literature [20, 21], patients notably favored the convenience of home dosing [23, 24]. Conversely, the most frequently cited reasons for patients transitioning from SCIG to fSCIG were the frequent dosing intervals associated with SCIG and local complaints. Despite the occurrence of local reactions with fSCIG, none of our patients cited them as a reason for discontinuing fSCIG. Patients emphasized that the less frequent dosing intervals associated with fSCIG were a crucial advantage of this treatment. Consistent with prior reports, the delivery of the drug via a single needle injection was identified as another factor favoring fSCIG over conventional SCIG applications [12, 23, 24].

Thus, fSCIG effectively combined the advantages of both IVIG and SCIG. In our study, although the maximum infusion volume reached per site with a single needle was above the maximum dose reported by Bauman et al., while it did not exceed the amount previously reported by Borte et al. [25, 26]. On the other hand, Wasserman et al. reported that maximum volume per infusion was expanded with multiple injection sites [12]. In another cohort of Polish pediatric patients, even larger target infusion volume was reported to be achieved with a ramp-up method [27]. Within the light of previous data, flexibility in infusion parameters and individualized application characteristics are emphasized in current data.

Over a 12-month period, two primary reasons for discontinuing fSCIG including mild systemic adverse reactions and difficulties in adhering to the procedures related to pump useage. While previous studies have mentioned local pain [1, 23, 26, 28], it's noteworthy that local reactions were not identified as a reason for treatment discontinuation in our cohort. For patients encountering local side effects post-administration, it was advised to divide the dose into multiple injections, administer smaller volumes with increased dosing intervals, and recline for 30 min to 1 h following treatment. Moreover, as it was recommended to our patients prior to injections, infusion at 2 fingers above the imaginary transverse umbilical line may be a proactive attitude to overcome local swelling, dispersion and switch from fSCIG due to these adverse reactions.

Following the transition from IVIG or SCIG to fSCIG, there was no significant change in the total Ig doses per 4 weeks among our study patients. The intervals for fSCIG infusions ranged from 2 to 4 weeks, with the majority of patients receiving the drug every 4 weeks, consistent with findings in the literature [23, 26, 29]. While the duration of fSCIG infusion was shorter than IVIG but longer than SCIG, we found that the median infusion duration was aligning with previous reports [30, 31]. The size and length of the needles were also similar to prior experience [24, 31]. Notably, all fSCIG administrations were conducted at a single site, with the abdominal region being the most common site, consistent with findings in the literature [1, 12, 24, 28, 31]. The absence of malnourished or overweight patients in our cohort is a determinant in our preference for SCIG. It has been discussed in the literature that patients with low fat content may tolerate subcutaneous treatment less, while obese patients may require longer needles, but conclusive evidence is lacking, and it has been concluded that each patient is evaluated individually [27].

Patients had serum IgG levels above the targeted IgG levels both before and during fSCIG, and no severe infections were observed. While there was no significant change in the frequency of major infections before and after fSCIG treatment in our cohort, in line with other studies [23, 28, 30, 32]. We observed a reduction in the frequency of upper respiratory tract infections following the initiation of fSCIG which may be due to reduction in fluctuations leading the control of minor infections. In accordance, the decrease in the number of minor infections was also reported to be in relation with maintaining a more stable Ig level which may contribute to controlling minor infections. [33]. Additionally, to our experience, there was a significant reduction in school/workdays missed among patients receiving fSCIG treatment which was related to ability for patients to receive treatment at home facilitated treatment planning.

Patients received the treatment safely, and no severe systemic adverse reactions were observed during the applications. The most common local adverse reaction in patients was swelling, with no decrease observed in repeated applications. Despite prevalent local reactions, none of the patients preferred treatment modification due to local adverse reactions. In terms of systemic adverse reactions, the most common were headache and one patient opted for a treatment modification due to headache and nausea, one patient due to fatigue. The absence of severe systemic reactions and the most common systemic adverse reaction of headache are consistent with the literature in which the frequency of local reactions has been reported to decrease with repeated administrations [26, 28, 31].

When assessing the quality-of-life, no significant difference in patients' quality of life was observed over the months. On the other hand, the assessment of patient treatment satisfaction was conducted using the TSQM-9 questionnaire, which was chosen for its omission of side effects section to potentially yield more objective results. In another cross-sectional survey with TSQM (version 1.4) including the subdomains related to side effects reported PID patients' medication satisfaction with fSCIG as highly satisfied with their treatments if score was > 73.53% [28]. In our study, a significant increase in treatment satisfaction was observed between baseline and sequential evaluation fSCIG treatment satisfaction which emphasizes the importance of evaluation in a continuous manner and demonstration of the improvement of treatment satisfaction over time. However, significant improvements were noted in the "effectiveness" subdomain in the surveys conducted every 3 months after patients started fSCIG treatment. In subgroup analyses, there was a significant improvement in effectiveness, baseline IVIG vs. fSCIG-receiving patients whereas a similar non-significant trend was observed for SCIG vs fSCIG. Significant improvements observed sequentially for all patients suggested that patients’ treatment satisfaction increased as their usage increased. Mallick et al. reported that a similar increase in effectiveness was observed in patients switching from IVIG to SCIG treatment, which was attributed to stable IgG levels rather than the variable IgG levels obtained with IV therapy [34].

Findings gathered from the current cohort demonstrate a high level of treatment satisfaction evaluated sequentially up to 12 months of fSCIG for PID patients. The multicenter nature of the cohort from different states, allowing for the evaluation of individuals from different social strata, and the longitudinal assessment of the quality of life and treatment satisfaction of both adults and children with PIDs resemble the uniqueness of the data. On the other hand, lack of per protocol data for all participants throughout the study may be accounted as a limitation of the study, whereas it can be presented as a real-life experience of fSCIG utilization as well.

In conclusion, fSCIG demonstrates admissible tolerability and efficacy in managing PIDs. Despite the notable frequency of local reactions, the identified benefits support the continuation of this therapy. These advantages include selective convenience over alternative routes, such as a lesser dose frequency compared to SCIG and the absence of a need for intravenous access and hospitalization compared to IVIG.

## Data Availability

No datasets were generated or analysed during the current study.
